# Impacts of coordinative training on normal weight and overweight/obese children’s attentional performance

**DOI:** 10.3389/fnhum.2015.00577

**Published:** 2015-10-28

**Authors:** Maria Chiara Gallotta, Gian Pietro Emerenziani, Sara Iazzoni, Marco Meucci, Carlo Baldari, Laura Guidetti

**Affiliations:** ^1^Department of Movement, Human and Health Sciences, University of Rome “Foro Italico”Rome, Italy; ^2^Department of Health and Exercise Science, Appalachian State UniversityBoone, NC, USA

**Keywords:** physical exercise, concentration, attention, school context, weight status

## Abstract

This study investigated the efficacy of a physical activity (PA) program to improve the attention span in normal weight vs. overweight/obese children. The study was designed as a cluster-randomized controlled intervention. One hundred fifty-seven normal weight and overweight/obese primary school children were randomly divided in three cohorts: Traditional PA, Coordinative PA and a Control group (not attending any PA). Before and after 5 months of intervention, children were administered the d2-R test of attention. Results showed that participants’ attentional performances were significantly affected by Time (pre vs. post; *P* < 0.01) and by Time × Group interaction (traditional vs. coordinative vs. control; *P* < 0.001), revealing significant different effects of intervention/exercise modality on children’s attentional performance, independently of their weight status. These data suggest that a 5-month school-based PA intervention can improve the cognitive performance in children. Further, the Coordinative PA intervention resulted in the most significant improvement in children’s attention.

## Introduction

The prevalence of overweight and obese children has risen progressively over the last two decades. In Europe, 20% of children (to 16 years of age) are overweight and 33% are obese (van der Kruk et al., 2013). While conflicting studies exist regarding the role of physical activity in weight loss (Luke and Cooper, 2013), a sedentary lifestyle has been shown to be the main contributor of obesity and obesity may lead to decrements in cognitive performance (Shore et al., 2008; Crova et al., 2014). The relationship between an increase in hypokinetic diseases and poor cognitive performance in children may be explained by the combination of socio-environmental factors (e.g., parental education, home environment) and biological factors (e.g., smaller gray matter volume, impaired cognitive functions by cytokines released from adipose tissue; Maayan et al., 2011; Veena et al., 2014).

Physical activity (PA) has been shown to improve cardiovascular fitness, lean mass and mental health in children (Meucci et al., 2013a,b). Amongst the mechanisms by which PA improves the aforementioned indices are resultant of neural stimulation (Davis et al., 2011) promoting biological adaptations combined with increases in brain neurotransmitters and cerebral blood flow which lend to tighter regulation of neurotrophins (Ploughman, 2008; Davis and Lambourne, 2009; Niederer et al., 2011). In addition to physical indicators, previous studies report that PA positively affects children’s mental health (Biddle and Asare, 2011) and cognitive functioning (Davis et al., 2011; Fedewa and Ahn, 2011; Tomporowski et al., 2011) leading to improvements in cognition (Tomporowski et al., 2008; Diamond and Lee, 2011). Attention and concentration are two aspects of cognitive functions that assume particular relevance during child development, as they are key elements in the learning process (Zervas and Stambulova, 1999). Specifically, selective and sustained attention is crucial for students that need to focus on a task and at the same time filter the environmental distractions (Semrud-Clikeman and Kutz, 2005). Developing these aspects is a cornerstone in both cognitive and emotional self-regulation and, therefore, translates to academic success by increasing goal-oriented behaviors, school readiness and scholastic success (Blair and Diamond, 2008).

The long-term effects of physical exercise on children’s cognitive functions have been widely studied by investigators by manipulating the quantitative aspects of physical exercise such as frequency and length of the training sessions (Voss et al., 2010; Davis et al., 2011). However, little is known regarding the qualitative aspects of the exercise intervention and their effects on children’s attention and concentration capacity (Voss et al., 2011; Pesce, 2012). With the term “qualitative aspects” of PA we refer to the type of PA, the complexity of movement and the variation of motor tasks and/or environmental factors (Mechling, 1999). Coordinative PA combines physical activity with attention, verbal learning and working memory (Budde et al., 2008). The school setting is considered an ideal setting since PA interventions are part of the education process.

Previous studies have randomized their subjects by age into low and higher intensity exercise groups. These studies demonstrated that acute bouts of coordinative exercises improved the attention span in normal-weight children aged 8–11 years (Gallotta et al., 2012) and adolescents aged 13–16 years (Budde et al., 2008), and that long-term coordinative exercises have positive effects on the executive function in kindergarten aged children (Chang et al., 2013). Improvements have been attributed to the activation of specific neuronal structures common to cognition and motor coordination (Budde et al., 2008) assuming a possible link between coordinative exercise and cognition (Chang et al., 2013). Specifically, the activation of the prefrontal cortex during movements with high complexity may be the possible mechanism responsible for the relationship between coordinative exercise and cognition. Coordinative exercises included complex movements involving multiple interactions between different body parts or with different objects (Egan et al., 2007) to develop psychomotor competence and expertise in movement-based problem solving. Accurate timing, temporal estimations, temporal production, and spatial adjustments are essential parts of the cognitive requirements (Buscà et al., 2011) to provide a constructive basis for improved cognitive performances. Current physical education in Italy offers constant movement combined with supervised activities that are meant to improve cognitive control (e.g., memory, speed of reaction, attention and concentration) while facilitating the learning process. Complex coordinative exercises may provide effective stimuli for prefrontal cortex development and consequently for the development of cognitive function in children. A paucity of literature exists regarding the effects of qualitative aspects of exercise on cognitive performance resultant of PA in overweight/obese children.

Therefore, the purpose of this study was to evaluate the effect of a qualitative PA intervention on sustained attention performance in overweight and obese children using two different exercise programs: a traditional PA intervention and a coordinative PA intervention. Since the execution of complex and coordinated movements involves the cerebellum and the prefrontal cortex (Budde et al., 2008; Best, 2010), we hypothesized that overweight/obese children will show increased cognitive performance in the coordinative group when compared with the traditional PA group.

## Materials and Methods

### Study Design and Setting

The study was designed as a cluster-randomized controlled intervention in all classes (from Grade 3 to Grade 5) of three primary schools in a rural area located about 50 km north of the city of Rome (Italy). The unit of randomization was the participating school. Thirteen classes with a total sample of 230 children between 8 and 11 years of age volunteered to participate in this study.

### Participants

The exclusion criteria were as follows: learning and academic difficulties, attention-deficit disorders, neurological and developmental disorders, dyslexia, medical conditions that would affect study results or limit physical activity.

The classroom demographics broke down to 88 Grade 3 children (8–9 years of age), 72 Grade 4 children (9–10 years of age) and 70 Grade 5 children (10–11 years of age). After cluster randomization, 78 participants were in the Traditional PA group, 83 participants in the Coordinative PA group, and the remaining 69 participants in the Control group (not attending any PA program).

Children were classified as normal weight (NW) or overweight/obese (OB) children in relation to their body fat mass percentage (FM%) according to the McCarthy’s age-sex specific cut-offs (McCarthy et al., 2006). Specifically, children were subdivided into two groups [NW: normal weight (2nd centile < FM% < 85th centile); OB: overweight/obese (FM% > 85th centile)]. Underweight children were excluded from the analyses, therefore, the final sample consisted of 156 primary school students with 56 children (33 normal weight and 23 overweight/obese) in the Traditional PA group, 59 children (40 normal weight and 19 overweight/obese) in the Coordinative PA, and 41 children (30 normal weight and 11 overweight/obese) in the Control group.

The University Ethical Committee approved this investigation in accordance with the ethical standards laid down in the 1964 Declaration of Helsinki and its later amendments (Rif 3502 Prot. 1883/15). Written informed consent and assent was obtained from both parents and children prior to study participation.

### Anthropometric Measurements

Pre- and post-intervention anthropometric measurements were used to assess children’s weight, height, BMI and body fat. Weight and height were measured using a scale and a stadiometer to the nearest 0.5 kg and 0.1 cm, respectively. Children’s body mass index (BMI) was calculated as weight in kg divided by the square of height in meters. Body fat percentage was measured by multi-frequency bioelectrical impedance analysis (IOI 353).

### Intervention

The intervention period lasted 5 months. Both PA interventions differed in type and mode of physical activities in which children were engaged but they were equivalent in structure, overall duration and intensity, and consisted of two 1 h sessions per week. The exercise intensity of both PA programs was monitored using an OMNI scale (Robertson et al., 2006) to avoid possible differences in intensity between the two types of PA programs.

PA interventions were designed by a Physical Educator who supervised one of the two weekly lessons; the other was conducted by the classroom teacher. The two PA interventions had the same structure, they included 15 min of warm-up, 30 min of moderate-to-vigorous physical activities (MVPA) within a range of 5 < RPE < 8 (Nelson et al., 2007), and 15 min of cool-down and stretching.

The traditional PA intervention was designed to promote health, fitness, sensory-motor, social and communicative development (Decree of the President of the Republic of Italy n. 254 of 16 November 2012, 2013). It was primarily focused on endurance, strength, flexibility exercises and circuit training for cardiovascular health (Ministry of Education, University and Research (MIUR), 2012). For more details, see the appendix.

The coordinative PA intervention was focused on improving the coordination and dexterity of the participants. It was organized in four different didactic modules lasting 5 weeks each one. Each module focuses on specific coordination abilities found in sports games, rhythmic activities, gymnastics and/or fitness activities. These activities were previously published by our lab (Gallotta, 2014; Gallotta et al., 2014).

The *sports games module* allowed children to recognize and manage the characteristics of traditional sports games and/or pre-sports (e.g., handball, mini-volleyball, mini-basketball). The educational proposals focused on specific aspects of games: rules, roles, spaces, times and strategies.

The *rhythmic activities module* was planned to specifically develop rhythmic and time perception abilities. Exercises or movement sequences were proposed, with or without tools, using wide execution variability in relation to the perception of some concepts such as “before”, “after”, “contemporary”, “next”, “slow”, “fast” and “cadence”. Sounds and/or music tracks were used.

The *gymnastics module* was characterized by a general movement development. Children were able to become aware of their movement patterns. Therefore, activities were proposed to allow children to be able to manage and vary the movement patterns as a function of spatial and temporal parameters, in executive situations of growing complexity.

The *fitness activities module* was planned to develop children’s strength, endurance, speed and flexibility.

The intensity of Coordinative PA intervention gradually increased in terms of complexity and degree of difficulty (concerning movement time, precision, amplitude, size of the target). The intervention was based on the systematic variation of practice by making task demands progressively more difficult. For more details, see the appendix.

### Experimental Measures

Before and after the intervention period, children completed the d2-R test of attention (Brickenkamp et al., 2013) to evaluate sustained attention and concentration under stress induced by completion time. The d2-R test is a paper-and-pencil letter cancellation test that consists of 14 different lines, each one composed of 47 randomly mixed letters (p, d), with one to four single and/or double quotation marks, either over and/or under each letter. Children were required to mark, within 20 s for each line, only the letters “d” that have double quotation marks either above or below them. The test lasted 4.67 min. Each child’s score was determined by the total number of items processed within the d2-R test, by the number of letters correctly marked minus errors of commission, and by the percentage of errors (% errors) made within all items processed. The total number of items processed was a measure of processing speed and amount of work completed, the number of letters correctly marked minus errors of commission was a measure of concentration performance, and % errors was a measure of performance quality. A low error rate indicated a more successful performance. The range of the test reliability was between 0.95 and 0.98, and the validity coefficient was 0.47 (Brickenkamp et al., 2013).

The d2-R test determines the capacity to focus on one stimulus/fact, while suppressing awareness to competing distractors (Brickenkamp et al., 2013). Selective attention was also required for successful completion since the letter “d” was not only orthographically similar to the letter “p” but there were several distractors within the text that the subject had to identify. The performance on this test reflects visual perceptual speed and concentration capacities.

### Statistical Analysis

All results were expressed as mean ± SD. Individual scores (items processed, % errors, concentration) were analyzed using a 3 × 2 × 2 mixed analysis of covariance (ANCOVA) with Group (Traditional PA group vs. Coordinative PA group vs. Control group), Time (pre vs. post), Weight Status (normal weight vs. overweight/obese children) as factors and baseline data as covariates. Effect size was also calculated using Cohen’s definition of small, medium, and large effect size (as partial *η*^2^ = 0.01, 0.06, 0.14). Significant interactions were further analyzed by means of Bonferroni *post hoc* analysis. Within the weight status factor, differences in the baseline attentional variable scores of normal weight and overweight/obese children were verified using an unpaired comparison *t*-test. Moreover, for each variable score evaluated after the intervention, the absolute variation (Δ) and the percentage of variation (Δ%) with respect to its pre-intervention value (post-intervention value − pre-intervention value) were calculated. An ANOVA was then performed to examine the effect of Group on absolute variation and on percentage of variation in each attentional variable, followed by *post hoc* analysis (Bonferroni adjustment) to determine effects within the three groups. Anthropometric data were analyzed using a 3 × 2 × 2 mixed-model repeated-measures analysis of covariance (ANCOVA) with Group (Traditional PA group vs. Coordinative PA group vs. Control group), Weight Status (normal weight vs. overweight/obese children), and Time (pre vs. post) as factors, with height included as covariate. Statistical significance was defined as *P* ≤ 0.05.

## Results

Anthropometric data are reported in Table 1. No significant effects were found for weight, BMI and %FM after intervention.

**Table 1:** **Pre- and post-intervention anthropometric characteristics of NW and OB children (mean values ± SD)**.

	NW		OB	
	Pre	Post	Pre	Post
**Traditional PA Group**
Weight (Kg)	36.0 ± 7.7	36.4 ± 7.5	51.2 ± 10.1	52.2 ± 10.1
Height (cm)	136.7 ± 9.2	137.9 ± 9.1*	141.4 ± 8.7	142.9 ± 8.9*
BMI (Kg/m^2^)	19.1 ± 2.1	18.9 ± 2.2	25.3 ± 2.4	25.3 ± 2.4
% FM	20.0 ± 3.6	19.7 ± 3.9	30.0 ± 3.8	30.0 ± 3.8
**Coordinative PA Group**
Weight (Kg)	36.7 ± 6.3	37.7 ± 6.7	48.6 ± 5.3	49.9 ± 5.6
Height (cm)	138.6 ± 7.5	138.9 ± 7.7	139.1 ± 7.1	140.2 ± 7.3*
BMI (Kg/m^2^)	19.2 ± 2.1	19.5 ± 2.2	25.1 ± 2.0	25.4 ± 2.1
% FM	19.9 ± 4.2	20.7 ± 4.2	29.8 ± 3.5	30.2 ± 3.5
**Control Group**
Weight (Kg)	34.3 ± 8.6	35.4 ± 8.9	57.2 ± 7.5	58.8 ± 7.8
Height (cm)	136.8 ± 9.0	137.6 ± 9.3*	144.5 ± 6.3	145.5 ± 6.6*
BMI (Kg/m^2^)	18.2 ± 2.7	18.5 ± 2.9	27.4 ± 3.1	27.7 ± 3.3
% FM	18.8 ± 2.5	19.5 ± 3.1	30.4 ± 3.9	30.7 ± 4.4

*P < 0.05 Pre vs. Post.

Table 2 reports only significant results that are relevant for the present study: main effect of Time and Time × Group Interaction. Children significantly improved their total number of items processed, concentration and % errors individual values after intervention (*P* < 0.001). A Time × Group Interaction indicates the likely presence of differential effects of intervention type on all variables following the intervention. No differential effects were reported for weight status.

**Table 2:** **Significant ANCOVA results on attentional test scores**.

Variable	Factors	*F*	df	*P*	Partial *η*^2^
**Items processed**	Time	96.73	1	0.000	0.394
	Time × Group	5.42	2	0.005	0.068
**Concentration**	Time	96.22	1	0.000	0.392
	Time × Group	10.89	2	0.000	0.128
**% errors**	Time	53.04	1	0.000	0.251
	Time × Group	16.90	2	0.000	0.185

Table 3 reports pre- and post-intervention individual scores. Total number of items processed significantly improved after intervention only in Traditional PA Group, while concentration and % errors values significantly improved after intervention in both Traditional and Coordinative PA Groups. No significant differences were observed for Control Group.

**Table 3:** **Pre- and post-PA intervention individual scores (mean ± SD) for items processed, concentration, and % errors**.

	Traditional PA Group		Coordinative PA Group		Control Group	
	Pre	Post	Pre	Post	Pre	Post
**Items processed**	375.09 ± 85.19	435.57 ± 87.41*	407.44 ± 95.79	399.70 ± 85.42	382.49 ± 92.41	396.85 ± 87.17
**Concentration**	132.73 ± 32.28	161.48 ± 38.43*	104.44 ± 42.51	144.63 ± 34.20*	123.07 ± 39.69	123.07 ± 40.74
**% errors**	6.87 ± 3.87	5.36 ± 5.06*	16.90 ± 8.29	5.89 ± 3.19*	10.82 ± 6.50	10.03 ± 5.19

*P ≤ 0.005 Pre vs. Post.

**Figure 1:**
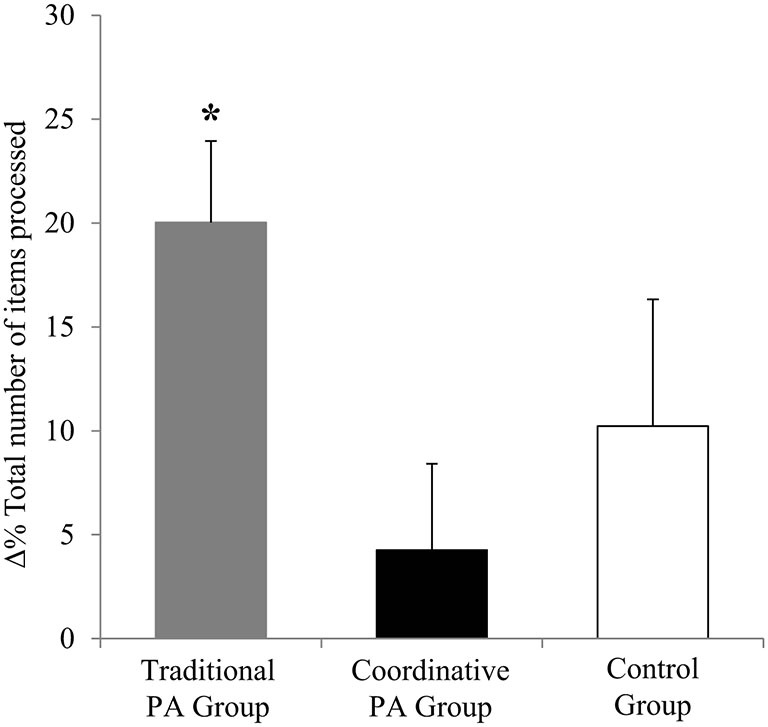
**Percentage of variation (Δ%) of the total number of items processed (± SEM).** *P < 0.05 Traditional PA Group vs. Coordinative PA Group.

**Figure 2:**
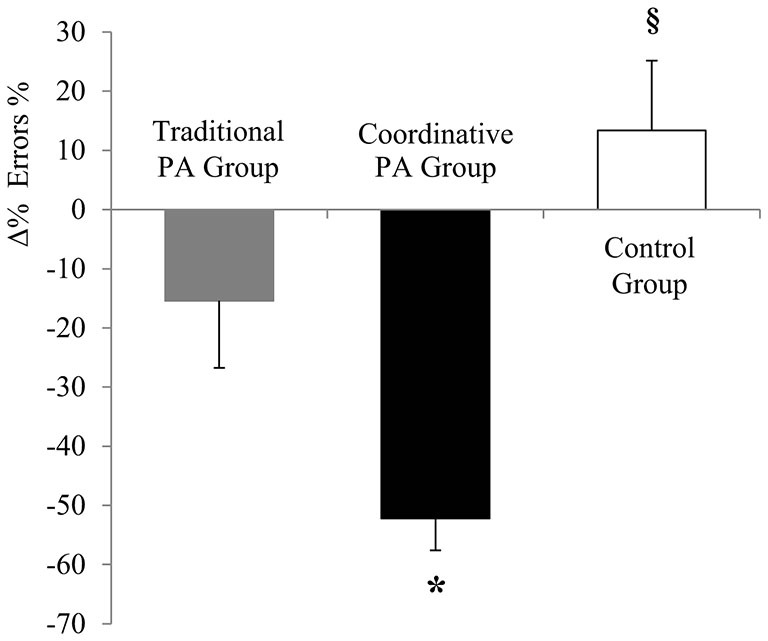
**Percentage of variation (Δ%) of the percentage of errors (% errors) (± SEM).** *P < 0.05 Coordinative PA Group vs. Traditional PA Group; ^§^*P* < 0.05 Control Group vs. Coordinative PA Group vs. Traditional PA Group.

**Figure 3:**
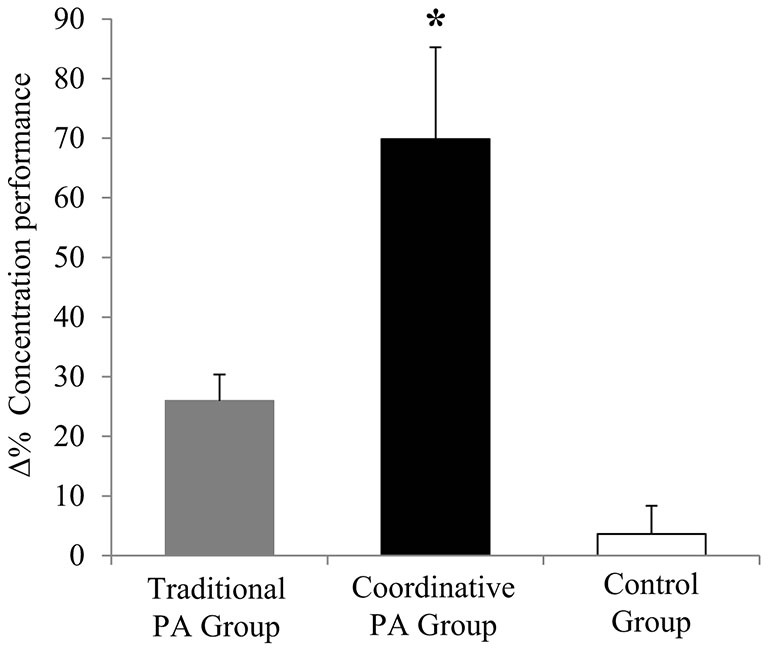
**Percentage of variation (Δ%) of concentration performance (± SEM).** *P < 0.05 Coordinative PA Group vs. Traditional PA Group vs. Control Group.

Improvements across the intervention were analyzed using Δ and Δ%. An ANOVA revealed a significant main effect of Group on Δ items processed (*F*_2,156_ = 4.65, *P* = 0.01, *η*^2^ = 0.063), on Δ concentration (*F*_2,156_ = 8.14, *P* = 0.0004, *η*^2^ = 0.098) and on Δ % errors (*F*_2,156_ = 28.8, *P* < 0, 0001, *η*^2^ = 0.281). Moreover, the ANOVA revealed a significant main effect of Group on Δ% items processed (*F*_2,156_ = 3.24, *P* = 0.04, *η*^2^ = 0.044), on Δ% concentration (*F*_2,156_ = 7.002, *P* = 0.001, *η*^2^ = 0.087), and on Δ% errors% (*F*_2,156_ = 9.58, *P* = 0.0001, *η*^2^ = 0.114). Traditional PA intervention led to a higher improvement of total number of items processed, while Coordinative PA intervention led to a higher improvement of concentration and % errors values than Traditional PA intervention and Control Group (Figures 1–3).

## Discussion

Our main finding showed that coordinative PA intervention was most effective at improving both the level and quality of concentration. Additionally, overweight/obese and normal-weight children equally benefitted from the PA intervention.

Children in the Traditional PA group significantly increased their working speed after 5 months, while concentration and accuracy scores improved in both activity groups at the end of the intervention period. Our findings revealed that the type of intervention showed differential effects on attention variables measured independently of children’s weight status. In particular, results revealed that Traditional PA intervention led to higher total number of items processed compared with Coordinative PA intervention, while Coordinative PA intervention led to higher concentration and % errors values compared with Traditional PA intervention and Control Group (Figures 1–3).

In accordance with previous studies, children in both activity groups showed higher levels of attention after 5 months of intervention compared to those in the Control Group supporting the theory that PA can improve mental function in children (Tomporowski et al., 2008, 2011). Davis et al. (2011) concluded that a direct path exists between exercise and cognitive performance, which may be responsible for the neural stimulation in response to movement. Currently, three hypotheses explain how exercise may augment cognitive control functions: (1) regulation of neurotrophins (such as growth factors); (2) increase in oxygen saturation due to increased blood flow and circulatory angiogenesis; and (3) increase in brain neurotransmitters (e.g., norepinephrine and serotonin) facilitating information processing (Ploughman, 2008; Niederer et al., 2011). The combination of these biological mechanisms stemming from an increase in exercise induces brain activity changes explaining the increase in children’s cognitive functions (Davis and Lambourne, 2009).

The increase in cognitive function following exercise training may be attributed to alterations of brain structure and function resulting from molecular and cellular processes that support neuronal plasticity (Hillman et al., 2008). The improved attention rate observed in both physical activity groups of our study could be the result of physiological brain changes and enhanced neuroplasticity (Chaddock et al., 2011). Moreover, the participation in organized PA sessions could have also developed psychological traits like self-efficacy and mastery, contributing to beneficial cognitive changes (Davis and Lambourne, 2009). Structured and complex sports and leisure activities that involve motor learning, interactions with the environment and group cooperation require mental operations which likely improve several cognitive processes such as attention and concentration capacity.

It is well known that exercise modality is a significant moderator of the exercise-cognition relation (Lambourne and Tomporowski, 2010; Hung et al., 2013) and that both acute and chronic exercise (Chang et al., 2013) with coordinative and attention demands may improve cognitive performance (Chang et al., 2013). The aforementioned author postulated that a link between coordinative exercise and cognitive functions may exist, stressing the prominent role of the coordinative exercise on executive functions in kindergarten children (Chang et al., 2013). Specifically, the activation of the prefrontal cortex during movement of high complexity seemed to explain the possible mechanism responsible for the relationship between coordinative exercise and cognition. Executive functions are strongly associated with the neural circuitry of the prefrontal cortex (Best, 2010) and complex motor activity execution is strongly associated with the activation of this region. Therefore, complex coordinative exercises could be effective stimuli for children’s prefrontal cortex development and for their cognitive functions development (Chang et al., 2013). This would likely explain the link with overweight and obese children and the association with poor cognitive performances (Shore et al., 2008; Crova et al., 2014).

To our knowledge, this is the first study that has analyzed the effect of different exercise protocols on concentration and attention capacity of overweight and obese children in a school setting. We hypothesized greater beneficial effects of both PA interventions in overweight/obese children compared to their normal-weight peers. Yet, a selective improvement of children’s attentional performance in the Coordinative PA Group when compared with Traditional PA Group. However, results revealed no group differences in attentional performance between obese and normal-weight children. The absence of significant differences at baseline between normal-weight and obese children on the measured attentional variables indicates that all children benefit from physical exercise, independently of their weight status. It was also possible that all children were quite familiar with the d2-R test of attention thanks to the relative ease of transfer from an academic class to this pen-and-paper test. However, our results were in contrast with those reported by Cserjési et al. (2007) who found that obese schoolboys performed worse on the d2 test of attention when compared with their normal-weight peers and suggested that childhood obesity implicates cognitive deficits in attentional performance (Cserjési et al., 2007). This difference could be explained by the fact that our participants presented with little differences in weight status between overweight/obese and normal-weight children (25.7 ± 2.6 vs. 18.9 ± 2.4 kg/m^2^, respectively) compared to the children studied by Cserjési et al. (2007) (27.2 ± 1.8 vs. 16.9 ± 1.3 kg/m^2^, respectively). Also, the negative relationship between adiposity and cognitive control was found in preadolescent children with higher %FM (Kamijo et al., 2012) which is not indicative of our sample.

Our second hypothesis was partially supported because children of Coordinative PA Group showed significant improvements in concentration capacity and percentage of errors without improvements in total number of items processed. These results suggest that coordinative exercises may have a predominant effect on children’s concentration capacity and on test execution accuracy rather than on processing speed performance. A previous study reported opposite results, revealing that 50 min of a coordinative PA lesson for children produced lower cognitive scores than a traditional PA or academic lesson (Gallotta et al., 2012). However, one variable that was not consistent between the two studies was the use of a stressor, which was produced by the combination of cognitive and physical tasks. Activities demanding high coordination seem to be effective for the development of executive functions, as the cognitive load necessary to perform complex movements is essential to produce the neuroplastic changes underlying skillful movements (Pesce, 2012). Coordinative exercises could specifically rely on prefrontal-dependent tasks in a child’s immature brain state by increasing attentional resources and enhancing the efficiency of neurocognitive processing (Chang et al., 2013). The positive effects on attentional performance could be due to the activation of some specific neuronal structures (cerebellum and prefrontal cortex) common to cognition and motor coordination (Budde et al., 2008). This is consistent with literature showing the relationship between motor coordination and cognitive performance (Kwok et al., 2011; Hötting et al., 2012; Chang et al., 2013). Neuromotor abilities in both peripheral (e.g., neuromuscular ability) and central nervous systems (e.g., brain neurocircuit) provide a possible constructive basis for improved cognitive performances. Simply, the more complex the motor task, the greater the benefit on cognitive performance (Davis and Lambourne, 2009). Black et al. (1990) reported that rats involved in complex exercises developed new neural connections within the cerebellum contrarily to those involved in simple running exercises. Our subjects were all children and a study by Hirtz et al. (1985) showed that prepubertal children are particularly susceptible to external stimuli (especially with regard to coordination) that influence brain function and coordination ability. Brain structure and neuronal systems are in a state of dedifferentiation (Davis et al., 2007) and coordinative PA may play a fundamental role in neural and cognitive development, increasing both motor coordination and cognitive functions. For this reason, we believe it is essential to stimulate prepubertal children with both coordinative- and cognitive- based activities. In the current study, we used the d2-R test performance to measure response inhibition and executive function (Reinert et al., 2013). The prefrontal cortex, closely linked with executive functions and movement coordination, could be the last region of the brain to mature, and therefore, each dimension of executive functions could benefit from coordinative training.

Limitations of the study include the lack of assessment of additional neuropsychological functions beyond performance on the d2-R test of attention. Another limitation of the study is that there was no control for cognitive/attentional stimulation *per se* which seems to be a key aspect of coordinative PA. Further research is needed to evaluate the impact of each didactic module and how the progression effects children’s attentional performance.

In conclusion, our findings suggest that a 5-month school-based PA intervention has beneficial effects on cognitive performance in school-aged children. Moreover, it seemed that coordinative PA could help children to concentrate on academic/cognitive tasks improving test performance and accuracy and consequently, academic achievement. Future research is needed to investigate the link between different exercise protocols and their effects on school achievement.

## Conflict of Interest Statement

The authors declare that the research was conducted in the absence of any commercial or financial relationships that could be construed as a potential conflict of interest.

## Appendix

### Description of a Traditional PA Lesson

The traditional PA lesson consisted of continuous aerobic circuit training followed by a sub-maximal shuttle run exercise. This lesson was focused on the improvement of cardiovascular endurance by performing different types of gaits (e.g., fast walking, running, skipping) without any specific coordinative request. The traditional PA lesson provided changes in executive modalities and some variations of intensity.

### Description of a Coordinative PA Lesson

The coordinative PA lesson consisted of the sport-unspecific use of basketballs in the context of mini-games. The basketballs were used in unconventional ways with varying game rules (e.g., use of foot-eye coordination techniques with basketballs). This lesson was geared toward the development of both motor control and perceptual-motor adaptation abilities. The high variability in the activities aimed to contribute to a multilateral development of coordinative abilities. This lesson was focused on the development of psychomotor competences and expertise in movement-based problem solving through functional use of a common tool (e.g., basketball), and considering various tasks that involved decision-making motor tasks and manipulative ball handling skills (e.g., bouncing, throwing, receiving a ball, and their combination). The coordinative PA lesson resulted from the combination of physical load due to the practice of physical exercises and of cognitive load requested to perform movement-based problem solving and decision-making tasks requiring accurate timing, temporal estimations, temporal production, spatial and temporal adjustments, and spatial and temporal orientation that were essential parts of the cognitive requirements to perform such a kind of activities. The coordinative PA lesson aimed to develop both motor control abilities and perceptual-motor adaptation abilities, by combining demands on gross-motor and manipulative control abilities and perceptual-motor adaptation abilities (particularly kinaesthetic differentiation and response orientation).
